# Dr. Dexter R. Belding

**Published:** 1912-07

**Authors:** 


Dr. Dexter R. Belding of Malone, N. Y., died May 18, aged 66. He was a graduate of Cleveland University of Medicine and Surgery, 1869.

